# A systematic review of decision aids that facilitate elements of shared decision-making in chronic illnesses: a review protocol

**DOI:** 10.1186/s13643-017-0557-9

**Published:** 2017-08-07

**Authors:** Thomas H. Wieringa, Marleen Kunneman, Rene Rodriguez-Gutierrez, Victor M. Montori, Maartje de Wit, Ellen M. A. Smets, Linda J. Schoonmade, Gabriela Spencer-Bonilla, Frank J. Snoek

**Affiliations:** 1 Department of Medical Psychology, VU University Medical Center (VUMC), De Boelelaan 1117, 1081 HV Amsterdam, the Netherlands; 2 Amsterdam Public Health research institute, VU University Medical Center and Academic Medical Center, Amsterdam, the Netherlands; 3 Knowledge and Evaluation Research Unit, Mayo Clinic, 200 First street SW, Rochester, MN 55905 USA; 4 Division of Endocrinology, Department of Internal Medicine, “Dr. Jose E. González” University Hospital, Autonomous University of Nuevo Leon, Avenue Gonzalitos s/n, Mitras Centro and Avenue Francisco I. Madero, 66460 Monterrey, Nuevo Leon Mexico; 5 Department of Medical Psychology, Academic Medical Center (AMC), Meibergdreef 9, 1100 DD Amsterdam, the Netherlands; 6 Medical Library, VU University, De Boelelaan 1117, 1081 HV Amsterdam, the Netherlands

**Keywords:** Decision aids, Chronic illnesses, Shared decision-making

## Abstract

**Background:**

Shared decision-making (SDM) is a patient-centred approach in which clinicians and patients work side-by-side to decide together on the best course of action for each patient’s particular situation. Six key elements of SDM can be distinguished: situation diagnosis, choice awareness, option clarification, discussion of harms and benefits, deliberation of patient preferences and making the decision. Decision aids (DAs) are tools that facilitate SDM. The impact of DAs for chronic illnesses on SDM, clinical and patient reported outcomes remains uncertain.

**Methods:**

We will perform a systematic review aiming to describe (a) which SDM elements are incorporated in DAs for adult patients with chronic conditions and (b) the effects of DA use on SDM, clinical and patient reported outcomes. This manuscript reports on the protocol for this systematic review. The following databases will be searched for relevant articles: PubMed, Embase, Web of Science, CINAHL and PsycINFO, from their inception to October 2016. We will ascertain ongoing research by querying experts and searching trial registries. To enhance feasibility, we will limit the review to randomized controlled trials (RCTs) including patients with chronic cardiovascular and/or respiratory diseases and/or diabetes. SDM elements incorporated in DAs, DA effects and DA itself will be described.

**Discussion:**

This study will characterize DAs for chronic illness and will provide an overview of their effects on SDM, clinical and patient reported outcomes. We anticipate this review will bring to light knowledge gaps and inform further research into the design and use of DAs for patients with chronic conditions.

**Systematic review registration:**

PROSPERO registration number: CRD42016050320.

**Electronic supplementary material:**

The online version of this article (doi:10.1186/s13643-017-0557-9) contains supplementary material, which is available to authorized users.

## Background

Shared decision-making (SDM) is a patient-centred approach in which clinicians and patients work together to choose the best course of action for each patient’s particular situation [1]. Although most SDM research has been conducted in the context of one-time decisions, SDM is also relevant in decisions that can be reconsidered over time, as is often the case in the self-management of chronic conditions [2].

In general, a distinction can be made between six key elements of SDM: situation diagnosis, choice awareness, option clarification, discussion of harms and benefits, deliberation of patient preferences and making the decision [1–4]. The opening of an SDM interaction involves a diagnostic conversation (situation diagnosis) [1]. This conversation focuses first on understanding the patient’s situation and establishing what aspects require action [1, 4]. When more than one reasonable alternative option is available, the clinician should clearly indicate this and highlight that the preferences of the patient are important in deciding on the course of action (choice awareness) [3]. Subsequently, the clinician and the patient discuss how each option fits and accommodates within each patient’s situation (option clarification, discussion of harms and benefits and deliberation of patient preferences). Finally, the clinician and patient reach a decision [2, 4]. When fruitful, SDM results in a course of action that is needed, wanted and more likely to be implemented [5, 6]. SDM may also help facilitate a stronger clinician-patient relationship and shared understanding of treatment of patients’ health and life goals [7, 8]. To date, the effects of SDM on clinical outcomes have been found to vary across studies [9–11].

To facilitate SDM, decision aids (DAs) have been developed for use by clinicians and patients, either in preparation for or during the clinical encounter [12, 13] and are designed to help them participate in decisions that involve weighing the harms and benefits of different treatment options [12]. DAs can increase patient knowledge, reduce decisional conflict, help patients choose an option that is congruent with their values, reduce the proportion of patients remaining undecided and/or who play a passive role in the decision-making process and can have a positive effect on patient-clinician communication [12, 14–17]. These findings, however, mostly relate to one-time decisions. Whether the DAs designed for use in chronic conditions actually support the key elements of SDM and improve outcomes is unclear.

The aims of this review therefore are to (1) describe which SDM elements are present in DAs for patients with chronic conditions, including cardiovascular diseases, chronic respiratory diseases and/or diabetes, (2) determine the effects of these DAs compared to usual care or active controls (i.e. alternative interventions such as patient education) on frequently studied SDM outcomes (i.e. decisional conflict, knowledge, patient participation in decision-making, treatment decision (preference), treatment satisfaction, decision satisfaction, conversation satisfaction, risk expectations and perceptions and consultation time) and (3) determine the effects of these DAs on clinical outcomes (i.e. lipid levels, blood pressure, smoking status, (maximal) oxygen uptake, glycaemic control, body mass index (BMI), adherence and achieving treatment goals) and patient reported outcomes (i.e. quality of life, perceived health status, emotional distress and self-efficacy) compared to usual care or active controls.

Since collecting data on DAs available for all chronic illnesses is unfeasible, we selected those chronic conditions the World Health Organization recognizes as most prevalent [18–20] and are most likely to require self-management. The selected SDM, clinical- and patient-reported outcomes are considered by the authors as most relevant for the selected chronic conditions. We hypothesize that DAs that cover multiple elements of SDM will be more likely to have positive effects on SDM (process) outcomes, as well as on patient reported outcomes. For clinical outcomes, we have no reason to hypothesize a consistent response.

## Methods

### Study design

This protocol adheres to the Preferred Reporting Items for Systematic Review and Meta-analysis Protocols (PRISMA-P) (see “Additional file 1 PRISMA-P checklist.pdf” for the PRISMA-P checklist) [21].

### Eligibility criteria

#### Type of studies

Articles will be selected if they report on randomized controlled trials (RCTs) comparing the use of DAs for one or more of the selected chronic conditions to usual care and/or active controls. There will be no limit to the study setting and time frame.

#### Type of participants

Studies enrolling adult (18 years or older) patients with a diagnosis of a chronic condition defined by the World Health Organization as main types [18–20] and requiring self-management: cardiovascular diseases (e.g. coronary heart disease, cerebrovascular disease, peripheral arterial disease, rheumatic heart disease, congenital heart disease, deep vein thrombosis, pulmonary embolism, myocardial infarction and stroke), chronic respiratory diseases (e.g. chronic obstructive pulmonary disease (COPD), asthma, occupational lung diseases and pulmonary hypertension) and/or diabetes (types 1 and 2) will be included.

#### Type of interventions

Any DA designed to help clinicians and/or adult patients in shared decision-making will be included [12].

#### Type of outcome measures

SDM outcomes (i.e. decisional conflict, knowledge, patient participation in decision-making, treatment decision (preference), treatment satisfaction, decision satisfaction, conversation satisfaction, risk expectations and perceptions and consultation time) will be assessed. Clinical outcomes (i.e. lipid levels (LDL cholesterol, HDL cholesterol, total cholesterol, triglycerides), blood pressure, smoking status, (maximal) oxygen uptake, glycaemic control, body mass index (BMI), adherence and achieving treatment goals) and patient reported outcomes (i.e. quality of life, perceived health status, emotional distress (anxiety, illness-related distress) and self-efficacy) will also be extracted. There will be no restrictions based on measurement methods.

### Information sources and search strategy

With the help of an expert librarian (LJS), we will design and conduct a search strategy to find eligible articles on RCTs in the following databases from inception to October 2016: PubMed, Embase, Web of Science, CINAHL (through EBSCO), PsycINFO (through EBSCO) and Cochrane Library (see Additional file 2 Search strategy.pdf for the search strategy). The design and conduction of this search strategy will be finished around October 2016. There will be no restrictions based on language, year of publication or year of development of the DAs. Around September 2017, the initial electronic search strategy will be carried out a second time for articles published between October 2016 and September 2017. This second electronic search strategy will be supplemented by screening the reference lists from included studies to identify potentially eligible studies that may have been missed. In addition, ongoing research will be traced by contacting experts in the field and searches in databases for ongoing research (including: http://isrctn.com, http://narcis.nl, http://trialregister.nl and http://www.clinicaltrials.gov). If published before the publication date of our systematic review (submission will take place around December 2017), ongoing studies will be included when data extraction for included studies is completed (September 2017). We will contact field experts to inquire about ongoing RCTs fulfilling our eligibility criteria. These contacts will be established through e-mail, Facebook, LinkedIn and other media or face-to-face contact in February 2017. Author contact will be documented by name of sender, date of contact and full content of e-mail, Facebook message, LinkedIn message or other way of contact. If multiple articles are available on one RCT, all will be included (articles on interim analyses as well). Search activities will be documented by filling in a table including search term(s), information source, date of coverage and total number of publications found.

### Data management

All search results will be uploaded into Covidence for automatic de-duplication (October 2016). Covidence will be used for both abstract (November and December 2016) and full-text screening (January 2017 until March 2017). The total number of results before and after de-duplication will be documented per database.

### Selection process

Prior to abstract screening, eligibility criteria will be iterated for clarity to ensure comprehension by reviewers. Two reviewers will independently assess whether the abstracts of articles meet eligibility criteria. Since some outcomes may not be reported in the abstract (e.g. due to word restrictions) but are in the full-text article, outcomes will not be considered during the abstract screening phase. When reviewers disagree about including an abstract, the full text will be considered. Abstract screening will take place from November to December 2016.

Following the screening of titles and abstracts, corresponding full-text articles will again be assessed independently by two reviewers. After a pilot with 20 included full-texts, discrepancies will be discussed and instructions and/or criteria adapted if needed. Disagreements and this phase will be resolved by consensus or arbitration by a third reviewer. Reasons for non-eligibility will be documented by the reviewers. Furthermore, agreement between reviewers (yes/no) and decision following consensus agreement (including date of consensus) will be captured for every reference. Chance-adjusted inter-rater agreement for full-text screening will be estimated using the Kappa statistic [22]. Full-text screening will take place from January 2017 to March 2017.

During both title/abstract and full-text screening, the total number of titles/abstracts or full-texts before and after screening will be documented, as well as the number of excluded titles/abstracts or full-texts (including reasons for exclusion of full-texts).

### Data collection process

Two reviewers will independently collect data for all eligible full-text articles on RCTs. Data will not be collected for articles on interim analyses, if articles on the same RCT based on the total follow-up period are available (we will include those with the total follow-up). Results for all time spans (follow-up measurements/time intervals) will be captured. If one DA is tested in multiple trials, all will be included. A data extraction form, including information about publication, DA characteristics, SDM elements and effectiveness, will be designed and pilot tested before use (see Additional file 3 Data to extract.pdf for the data extraction form). After extracting data from five full-text reports (or all articles when less than five full-text articles will be eligible), the noted differences between reviewers will be discussed in order to get optimal calibration for data extraction. If necessary or desired, the extraction form will be adapted based on feedback from the reviewers to improve usability and ensure completeness. Similar to article selection, two or more reviewers will independently extract data. Disagreements will be resolved by consensus. If consensus on data extraction between the two parties cannot be reached, a third reviewer will arbitrate.

A recent study showed that health information tools developed and tested online hardly remain available and accessible [23]. Therefore, all corresponding authors of included studies will be contacted through e-mail to assess whether the DA is currently available and used in practice. Non-responders will be sent a reminder email after 2 weeks. If the second attempt is unsuccessful, other authors will be contacted. If none of the authors responds, we will contact the corresponding author (or other authors) by phone. Every author contact will be documented by name of the sender, date of contact and full content of e-mail contact or a summary of telephone contact. See Additional file 3 for the characteristics per DA to be retrieved and entered in the data extraction form. Data collection will take place around August 2017.

### Missing data

If data presented in the studies is unclear, missing or presented in a form that is either un-extractable or difficult to reliably extract, we will request data from the authors following the same author contact protocol described above. As above, author contact will be documented by date and full content of e-mail contact.

### Risk of bias in individual studies

Risk of bias will be assessed in individual studies using the Cochrane Collaboration’s tool for assessing RCTs risk of bias. This tool takes into consideration six domains: (1) sequence generation, (2) allocation concealment, (3) blinding of participants and personnel, (4) blinding of outcome assessment, (5) incomplete outcome data, (6) selective outcome reporting and (7) other biases. Two reviewers will independently assess the risk of bias at all domains for every RCT [24]. Criteria for judgement per domain are to be found in Chapter 8 of the Cochrane Handbook for Systematic Reviews of Interventions [25]. Disagreement will again be resolved by consensus or if not possible, by arbitration of a third reviewer. Risk of bias in individual studies will enable a critical view on interpretation of DA effects found and will be assessed around September 2017.

### Outcomes and data synthesis

We will describe the RCTs included in our review, as well as the DAs that are tested in these studies. This includes the SDM elements incorporated in DAs, the effects of DAs on SDM outcomes (i.e. decisional conflict, knowledge, patient participation in decision-making, treatment decision (preference), treatment satisfaction, decision satisfaction, conversation satisfaction, risk expectations and perceptions, consultation time), clinical outcomes (i.e. lipid levels (LDL cholesterol, HDL cholesterol, total cholesterol, triglycerides), blood pressure, smoking status, (maximal) oxygen uptake, glycaemic control, body mass index (BMI), adherence and achieving treatment goals) and patient reported outcomes (i.e. quality of life, perceived health status, emotional distress (anxiety, illness-related distress) self-efficacy). For continuous outcomes mean (change) differences between intervention and control group, together with *p* values and 95% confidence intervals (95% CIs), will be extracted. Regarding dichotomous outcomes, both risk ratios (RRs) and odds ratios (ORs) with 95% CIs will be extracted or calculated if needed and possible. Furthermore, elements of SDM incorporated in DAs, risk of bias per RCT and DA itself will be described. Since heterogeneous populations and outcomes will be synthesized and much heterogeneity in time spans/intervals is expected, performing a meta-analysis will be difficult and perhaps not as useful. Therefore, in the likely event that conducting random-effects meta-analyses of the effects of these DAs on outcomes proves unwise, we will summarize the results narratively. Data will be synthesized around October 2017.

## Discussion

This is an overview of chronic care DAs developed and tested in RCTs, SDM elements they support and their effects on clinical and patient reported outcomes. The insights produced in it will help inform further research aimed at developing, testing and successfully implementing future DAs in clinical practice for patients with chronic conditions.

Our proposed review also has potential limitations. Other than duplicate assessment and clear eligibility criteria, we do not have safeguards in place to prevent a biased set of studies to be included. Also, since we are interested in the efficacy of DAs, we will limit our search strategy to RCTs as these have the most valid experimental design of research [26]. This may exclude (well designed and developed) DAs that have not (yet) been tested in trials. Finally, we limit our search strategy to the most prevalent cardiovascular diseases, chronic respiratory diseases and diabetes [18–20], an incomplete list of chronic diseases. Learnings from this review may help further study the utility of DAs in the SDM process in less prevalent chronic conditions.

This review will provide a broad overview of DAs available for patients with cardiovascular, chronic respiratory diseases and diabetes, as well as SDM elements they incorporate and their effects on a broad range of outcomes. It may bring to light useful information to a variety of stakeholders including funding agencies, policy-makers, researchers, clinicians and patients with chronic conditions with the objective of delivering kind and careful care to patients with chronic conditions.

## Additional files

Additional file 1: PRISMA-P-checklist. (PDF 44 kb)

Additional file 2: Search strategy. (PDF 82 kb)

Additional file 3: Data to extract. (PDF 176 kb)

## Abbreviations

95% CI: 95% confidence interval
DA: Decision aid
OR: Odds ratio
PRISMA-P: Preferred Reporting Items for Systematic Review and Meta-analysis Protocols
RCT: Randomized controlled trial
RR: Relative risk
SDM: Shared decision-making
