# Supplementary material for: A systematic review of decision aids that facilitate elements of shared decision-making in chronic illnesses: a review protocol
Source: Syst Rev. 2017 Aug 7;6:155. doi: 10.1186/s13643-017-0557-9 (PMC5545866; doi:10.1186/s13643-017-0557-9)
Supplement: Supplementary file 2 — Search strategy. (PDF 82 kb) [file 13643_2017_557_MOESM2_ESM.pdf]

Table of Contents

Pubmed 2

Embase 3

Cinahl 4

PsycINFO 6

Web of Science 8

Cochrane Library 9

Pubmed

1. **Decision**

"Decision Making"[Mesh] OR "Clinical Decision-Making"[Mesh] OR Decision*[tiab]

2. **Shared**

"Patient-Centered Care"[Mesh] OR "Patient Participation"[Mesh] OR "Patient Preference"[Mesh] OR share*[tiab] OR sharing[tiab] OR patient centered*[tiab] OR patient centred[tiab] OR patient focused[tiab] OR sdm[tiab] OR prefer*[tiab]

3. **Aids**

"Decision Support Techniques"[Mesh] OR "Decision Making, Computer-Assisted"[Mesh] OR tool*[tiab] OR aid[tiab] OR aids[tiab] OR intervention*[tiab] OR support*[tiab] OR instrument*[tiab]

4. **Chronisch zieken**

"Asthma"[Mesh] OR "Pulmonary Disease, Chronic Obstructive"[Mesh] OR asthma*[tiab] OR copd[tiab] OR chronic respiratory disease*[tiab] OR chronic obstructed pulmonary disease*[tiab] OR chronic obstructive airway disease*[tiab] OR chronic obstructive lung disease*[tiab] OR chronic bronchitis[tiab] OR emphysema[tiab] OR coad[tiab] OR chronic airflow obstruction*[tiab] OR "Diabetes Mellitus"[Mesh] OR diabetes[tiab] OR diabetic*[tiab] OR dm2[tiab] OR niddm[tiab] OR dm 2[tiab] OR t2d*[tiab] OR dm type 2[tiab] OR dm type II[tiab] OR dm1[tiab] OR iddm[tiab] OR dm 1[tiab] OR t1d*[tiab] OR dm type 1[tiab] OR dm type I[tiab] OR "Cardiovascular Diseases"[Mesh] OR cardiovascular disease*[tiab] OR cardiovascular disorder*[tiab] OR cardiovascular disturbance*[tiab] OR cardiovascular lesion*[tiab] OR cardiovascular syndrome*[tiab] OR cvd[tiab] OR myocardial ischem*[tiab] OR myocardial infarct*[tiab] OR heart disease*[tiab] OR coronary disease*[tiab] OR artery disease*[tiab] OR arterial disease*[tiab] OR heart attack*[tiab] OR heart failure*[tiab] OR cardiac failure*[tiab] OR high blood pressure*[tiab] OR hypertensi*[tiab] OR heart patient*[tiab] OR cerebrovascular disease*[tiab] OR cerebrovascular disorder*[tiab] OR vein thrombos*[tiab] OR embolism*[tiab] OR stroke*[tiab] OR cerebrovascular accident*[tiab] OR cva[tiab] OR cvas[tiab] OR vascular accident*[tiab] OR apoplexy[tiab] OR brain infarction*[tiab]

5. **Studiefilter**

(randomized controlled trial[pt] OR controlled clinical trial[pt] OR randomized controlled trials[mh] OR random allocation[mh] OR double-blind method[mh] OR single-blind method[mh] OR clinical trial[pt] OR clinical trials[mh] OR clinical trial*[tw] OR ((singl*[tw] OR doubl*[tw] OR trebl*[tw] OR tripl*[tw]) AND (mask*[tw] OR blind*[tw])) OR rct[tiab] OR intervention*[tiab] OR "latin square"[tw] OR placebos[mh] OR placebo*[tw] OR random*[tw] OR research design[mh] OR comparative study[pt] OR evaluation studies[pt] OR follow-up studies[mh] OR prospective studies[mh] OR cross-over studies[mh] OR control[tw] OR controll*[tw] OR prospectiv*[tw] OR volunteer*[tw]) NOT (animals[mh] NOT humans[mh])

Embase

1. **Decision**

'decision making'/exp OR 'clinical decision making'/exp OR decision*:ab,ti

2. **Shared**

'patient participation'/exp OR 'patient preference'/exp OR share*:ab,ti OR sharing:ab,ti OR ‘patient centered*’:ab,ti OR ‘patient centred’:ab,ti OR ‘patient focused’:ab,ti OR sdm:ab,ti OR prefer*:ab,ti

3. **Aids**

'decision support system'/exp OR tool*:ab,ti OR aid:ab,ti OR aids:ab,ti OR intervention*:ab,ti OR support*:ab,ti OR instrument*:ab,ti

4. **Chronisch zieken**

'asthma'/exp OR 'chronic obstructive lung disease'/exp OR asthma*:ab,ti OR copd:ab,ti OR ‘chronic respiratory disease*’:ab,ti OR ‘chronic obstructed pulmonary disease*’:ab,ti OR ‘chronic obstructive airway disease*’:ab,ti OR ’chronic obstructive lung disease*’:ab,ti OR ‘chronic bronchitis’:ab,ti OR emphysema:ab,ti OR coad:ab,ti OR ‘chronic airflow obstruction*’:ab,ti OR 'diabetes mellitus'/exp OR diabetes:ab,ti OR diabetic*:ab,ti OR dm2:ab,ti OR niddm:ab,ti OR ‘dm 2’:ab,ti OR t2d*:ab,ti OR ‘dm type 2’:ab,ti OR ‘dm type II’:ab,ti OR dm1:ab,ti OR iddm:ab,ti OR ‘dm 1’:ab,ti OR t1d*:ab,ti OR ‘dm type 1’:ab,ti OR ‘dm type I’:ab,ti OR 'cardiovascular disease'/exp OR ‘cardiovascular disease*’:ab,ti OR ‘cardiovascular disorder*’:ab,ti OR ‘cardiovascular disturbance*’:ab,ti OR ‘cardiovascular lesion*’:ab,ti OR ‘cardiovascular syndrome*’:ab,ti OR cvd:ab,ti OR ‘myocardial ischem*’:ab,ti OR ‘myocardial infarct*’:ab,ti OR ‘heart disease*’:ab,ti OR ‘coronary disease*’:ab,ti OR ‘artery disease*’:ab,ti OR ‘arterial disease*’:ab,ti OR ‘heart attack*’:ab,ti OR ‘heart failure*’:ab,ti OR ‘cardiac failure*’:ab,ti OR ‘high blood pressure*’:ab,ti OR hypertensi*:ab,ti OR ‘heart patient*’:ab,ti OR ‘cerebrovascular disease*’:ab,ti OR ‘cerebrovascular disorder*’:ab,ti OR ‘vein thrombos*’:ab,ti OR embolism*:ab,ti OR stroke*:ab,ti OR ‘cerebrovascular accident*’:ab,ti OR cva:ab,ti OR cvas:ab,ti OR ‘vascular accident*’:ab,ti OR apoplexy:ab,ti OR ‘brain infarction*’:ab,ti

5. **Studiefilter**

'intervention study'/exp OR 'clinical trial'/exp OR 'study design'/exp OR 'Latin square design'/exp OR 'comparative study'/exp OR 'controlled study'/exp OR 'crossover procedure'/exp OR 'double blind procedure'/exp OR 'randomized controlled trial'/exp OR 'single blind procedure'/exp OR 'prospective study'/exp OR 'comparative study'/exp OR 'evaluation study'/exp OR 'crossover procedure'/exp OR intervention*:ab,ti OR rct:ab,ti OR random*:ab,ti OR factorial*:ab,ti OR crossover*:ab,ti OR ‘cross over*’:ab,ti OR placebo*:ab,ti OR comparative:ab,ti OR comparing:ab,ti OR ‘evaluation stud*’:ab,ti OR trial*:ab,ti OR control*:ab,ti OR prospective:ab,ti OR assign*:ab,ti OR allocat*:ab,ti OR volunteer*:ab,ti OR ((singl*:ab,ti OR doubl*:ab,ti OR trebl*:ab,ti OR tripl*:ab,ti) AND (mask*:ab,ti OR blind*:ab,ti)) NOT ('animal'/exp NOT 'human'/exp)

Cinahl

1. **Decision**

MH ("Decision Making" OR "Decision Making, Clinical" OR "Decision Making, Patient") OR TI decision* OR AB decision*

2. **Shared**

MH ("Patient Centered Care" OR "Consumer Participation") OR TI (share* OR sharing OR patient centered* OR patient centred OR patient focused OR sdm OR prefer*) OR AB (share* OR sharing OR patient centered* OR patient centred OR patient focused OR sdm OR prefer*)

3. **Aids**

MH ("Decision Support Techniques+" OR "Decision Support Systems, Clinical" OR "Decision Making, Computer Assisted") OR TI (tool* OR aid OR aids OR intervention* OR support* OR instrument*) OR AB (tool* OR aid OR aids OR intervention* OR support* OR instrument*)

4. **Chronisch zieken**

MH ("Asthma+" OR "Pulmonary Disease, Chronic Obstructive+" OR "Cardiovascular Diseases+" OR "Diabetes Mellitus+" OR "Diabetes Mellitus, Type 2" OR "Diabetes Mellitus, Type 1+" OR "Diabetes Mellitus, Gestational") OR AB (asthma* OR copd OR “chronic respiratory disease*” OR “chronic obstructed pulmonary disease*” OR “chronic obstructive airway disease*” OR “chronic obstructive lung disease*” OR “chronic bronchitis” OR emphysema OR coad OR “chronic airflow obstruction*” OR diabetes OR diabetic* OR dm2 OR niddm OR “dm 2” OR t2d* OR “dm type 2” OR “dm type II” OR dm1 OR iddm OR “dm 1” OR t1d* OR “dm type 1” OR “dm type I” OR “cardiovascular disease*” OR “cardiovascular disorder*” OR “cardiovascular disturbance*” OR “cardiovascular lesion*” OR “cardiovascular syndrome*” OR cvd OR “myocardial ischem*” OR “myocardial infarct*” OR “heart disease*” OR “coronary disease*” OR “artery disease*” OR “arterial disease*” OR “heart attack*” OR “heart failure*” OR “cardiac failure*” OR “high blood pressure*” OR hypertensi* OR “heart patient*” OR “cerebrovascular disease*” OR “cerebrovascular disorder*” OR “vein thrombos*” OR embolism* OR stroke* OR “cerebrovascular accident*” OR cva OR cvas OR “vascular accident*” OR apoplexy OR “brain infarction*”) OR TI (asthma* OR copd OR “chronic respiratory disease*” OR “chronic obstructed pulmonary disease*” OR “chronic obstructive airway disease*” OR “chronic obstructive lung disease*” OR “chronic bronchitis” OR emphysema OR coad OR “chronic airflow obstruction*” OR diabetes OR diabetic* OR dm2 OR niddm OR “dm 2” OR t2d* OR “dm type 2” OR “dm type II” OR dm1 OR iddm OR “dm 1” OR t1d* OR “dm type 1” OR “dm type I” OR “cardiovascular disease*” OR “cardiovascular disorder*” OR “cardiovascular disturbance*” OR “cardiovascular lesion*” OR “cardiovascular syndrome*” OR cvd OR “myocardial ischem*” OR “myocardial infarct*” OR “heart disease*” OR “coronary disease*” OR “artery disease*” OR “arterial disease*” OR “heart attack*” OR “heart failure*” OR “cardiac failure*” OR “high blood pressure*” OR hypertensi* OR “heart patient*” OR “cerebrovascular disease*” OR “cerebrovascular disorder*” OR “vein thrombos*” OR embolism* OR stroke* OR “cerebrovascular accident*” OR cva OR cvas OR “vascular accident*” OR apoplexy OR “brain infarction*”)

5. **Studietypes**

MH ("Clinical Trials+" OR "Quantitative Studies" OR "Study Design+" OR "Random Assignment" OR "Evaluation Research" OR "Comparative Studies") OR (PT Clinical trial) OR (TX clini* N1 trial*) OR (TX ((singl* N1 blind*) OR (singl* N1 mask*)) OR TX ((doubl* N1 blind*) OR (doubl* N1 mask*)) OR TX ((tripl* N1 blind*) OR (tripl* N1 mask*))) OR (TX randomi* control*) OR ((TX random* allocat*) OR (TX allocat* random*)) OR (TX placebo*) OR (TX (waitlist* OR (wait* AND list*)) AND (control* OR group))) OR ((TX "treatment as usual") OR (TX tau)) OR (TX (control* N3 (trial* OR

study OR studies OR group*))) OR TX (rct OR intervention* OR “latin square” OR prospectiv* OR volunteer OR follow or factorial* OR crossover* OR “cross over”OR comparative OR comparing OR “evaluation stud*”)

PsycINFO

1. **Decision**

DE "Decision Making" OR TI decision* OR AB decision*

2. **Shared**

DE ("Client Participation") OR TI (share* OR sharing OR patient centered* OR patient centred OR patient focused OR sdm OR prefer*) OR AB (share* OR sharing OR patient centered* OR patient centred OR patient focused OR sdm OR prefer*)

3. **Aids**

DE "Decision Support Systems" OR TI (tool* OR aid OR aids OR intervention* OR support* OR instrument*) OR AB (tool* OR aid OR aids OR intervention* OR support* OR instrument*)

4. **Chronisch zieken**

DE ("Asthma" OR "Chronic Obstructive Pulmonary Disease" OR "Bronchial Disorders" OR "Pulmonary Emphysema" OR "Cardiovascular Disorders" OR "Aneurysms" OR "Arteriosclerosis" OR "Blood Pressure Disorders" OR "Cerebrovascular Disorders" OR "Embolisms" OR "Heart Disorders" OR "Hemorrhage" OR "Hypertension" OR "Ischemia" OR "Thromboses" OR "Diabetes" OR "Diabetes Insipidus" OR "Diabetes Mellitus" OR "Type 2 Diabetes" OR "Gestational Diabetes" OR DE "Blood Sugar") OR AB (asthma* OR copd OR “chronic respiratory disease*” OR “chronic obstructed pulmonary disease*” OR “chronic obstructive airway disease*” OR “chronic obstructive lung disease*” OR “chronic bronchitis” OR emphysema OR coad OR “chronic airflow obstruction*” OR diabetes OR diabetic* OR dm2 OR niddm OR “dm 2” OR t2d* OR “dm type 2” OR “dm type II” OR dm1 OR iddm OR “dm 1” OR t1d* OR “dm type 1” OR “dm type I” OR “cardiovascular disease*” OR “cardiovascular disorder*” OR “cardiovascular disturbance*” OR “cardiovascular lesion*” OR “cardiovascular syndrome*” OR cvd OR “myocardial ischem*” OR “myocardial infarct*” OR “heart disease*” OR “coronary disease*” OR “artery disease*” OR “arterial disease*” OR “heart attack*” OR “heart failure*” OR “cardiac failure*” OR “high blood pressure*” OR hypertensi* OR “heart patient*” OR “cerebrovascular disease*” OR “cerebrovascular disorder*” OR “vein thrombos*” OR embolism* OR stroke* OR “cerebrovascular accident*” OR cva OR cvas OR “vascular accident*” OR apoplexy OR “brain infarction*”) OR TI (asthma* OR copd OR “chronic respiratory disease*” OR “chronic obstructed pulmonary disease*” OR “chronic obstructive airway disease*” OR “chronic obstructive lung disease*” OR “chronic bronchitis” OR emphysema OR coad OR “chronic airflow obstruction*” OR diabetes OR diabetic* OR dm2 OR niddm OR “dm 2” OR t2d* OR “dm type 2” OR “dm type II” OR dm1 OR iddm OR “dm 1” OR t1d* OR “dm type 1” OR “dm type I” OR “cardiovascular disease*” OR “cardiovascular disorder*” OR “cardiovascular disturbance*” OR “cardiovascular lesion*” OR “cardiovascular syndrome*” OR cvd OR “myocardial ischem*” OR “myocardial infarct*” OR “heart disease*” OR “coronary disease*” OR “artery disease*” OR “arterial disease*” OR “heart attack*” OR “heart failure*” OR “cardiac failure*” OR “high blood pressure*” OR hypertensi* OR “heart patient*” OR “cerebrovascular disease*” OR “cerebrovascular disorder*” OR “vein thrombos*” OR embolism* OR stroke* OR “cerebrovascular accident*” OR cva OR cvas OR “vascular accident*” OR apoplexy OR “brain infarction*”)

5. **Study type**

DE "Treatment Effectiveness Evaluation" OR DE "Clinical Trials" OR DE "Placebo" OR TI (placebo* OR randomly) OR AB (placebo* OR randomly) OR TX randomi* OR TI trial OR AB trial OR TX ((singl* OR doubl* OR trebl* OR tripl*) N3 (blind* OR mask* OR dummy)) OR TI (control* N3 (trial* OR study OR studies OR group*)) OR AB (control* N3 (trial* OR study OR studies OR group*)) OR TI factorial* OR AB factorial* OR TI allocat* OR AB allocat* OR TI assign* OR AB assign* OR TI volunteer* OR AB volunteer* OR TI (crossover* OR "cross over*") OR AB (crossover* OR "cross over*") OR TX (quasi N5 (experimental OR random*)) OR AB (intervention* OR rct OR comparative

OR comparing OR “evaluation stud* OR prospective) OR TI (intervention* OR rct OR comparative OR comparing OR “evaluation stud* OR prospective)

Web of Science

1. **Decision**

TS = (Decision*)

2. **Shared**

TS=(share* OR sharing OR patient centered* OR patient centred OR patient focused OR sdm OR prefer*)

3. **Aids**

TS =(tool* OR aid OR aids OR intervention* OR support* OR instrument*)

4. **Chronisch zieken**

TS=(asthma OR "chronic obstructed pulmonary disease*" OR copd OR “chronic respiratory disease*” OR “chronic obstructive airway disease*” OR “chronic obstructive lung disease*” OR “chronic bronchitis” OR emphysema OR coad OR “chronic airflow obstruction*” OR diabetes OR diabetic* OR dm2 OR niddm OR “dm 2” OR t2d* OR “dm type 2” OR “dm type II” OR dm1 OR iddm OR “dm 1” OR “t1d*” OR “dm type 1” OR “dm type I” OR “cardiovascular disease*” OR “cardiovascular disorder*” OR “cardiovascular disturbance*” OR “cardiovascular lesion*” OR “cardiovascular syndrome*” OR cvd OR “myocardial ischem*” OR “myocardial infarct*” OR “heart disease*” OR “coronary disease*” OR “artery disease*” OR “arterial disease*” OR “heart attack*” OR “heart failure*” OR “cardiac failure*” OR “high blood pressure*” OR hypertensi* OR “heart patient*” OR “cerebrovascular disease*” OR “cerebrovascular disorder*” OR “vein thrombos*” OR embolism* OR stroke* OR “cerebrovascular accident*” OR cva OR cvas OR “vascular accident*” OR apoplexy OR “brain infarction*”)

5. **Studietypes**

TS = (rct OR random* OR control* OR trial OR placebo* OR compar* OR group OR groups OR therapy OR treatment OR intervention OR “research design” OR comparative OR “evaluation stud*” OR “follow-up stud*” OR prospective ” OR “single blind” OR “double blind” OR “trebl* blind” OR “triple blind” OR factorial OR allocat* OR assign* OR volunteer* OR crossover OR “cross over”) OR TI= (rct OR random* OR control* OR trial OR placebo* OR compar* OR group OR groups OR therapy OR treatment OR intervention OR “research design” OR comparative OR “evaluation stud*” OR “follow-up stud*” OR prospective ” OR “single blind” OR “double blind” OR “trebl* blind” OR “triple blind” OR factorial OR allocat* OR assign* OR volunteer* OR crossover OR “cross over”)

Cochrane Library

1. **Decision**

decision*

2. **Shared**

share* OR sharing OR patient centered* OR patient centred OR patient focused OR sdm OR prefer*

3. **Aids**

tool* OR aid OR aids OR intervention* OR support* OR instrument*

4. **Chronisch zieken**

asthma OR "chronic obstructed pulmonary disease*" OR copd OR “chronic respiratory disease*” OR “chronic obstructive airway disease*” OR “chronic obstructive lung disease*” OR “chronic bronchitis” OR emphysema OR coad OR “chronic airflow obstruction*” OR diabetes OR diabetic* OR dm2 OR niddm OR “dm 2” OR t2d* OR “dm type 2” OR “dm type II” OR dm1 OR iddm OR “dm 1” OR “t1d*” OR “dm type 1” OR “dm type I” OR “cardiovascular disease*” OR “cardiovascular disorder*” OR “cardiovascular disturbance*” OR “cardiovascular lesion*” OR “cardiovascular syndrome*” OR cvd OR “myocardial ischem*” OR “myocardial infarct*” OR “heart disease*” OR “coronary disease*” OR “artery disease*” OR “arterial disease*” OR “heart attack*” OR “heart failure*” OR “cardiac failure*” OR “high blood pressure*” OR hypertensi* OR “heart patient*” OR “cerebrovascular disease*” OR “cerebrovascular disorder*” OR “vein thrombos*” OR embolism* OR stroke* OR “cerebrovascular accident*” OR cva OR cvas OR “vascular accident*” OR apoplexy OR “brain infarction”

5. **Studietypes**

rct OR random* OR control* OR trial OR placebo* OR compar* OR group OR groups OR therapy OR treatment OR intervention OR “research design” OR comparative OR “evaluation stud*” OR “follow-up stud*” OR prospective OR “single blind” OR “double blind” OR “trebl* blind” OR “triple blind” OR factorial OR allocat* OR assign* OR volunteer* OR crossover OR “cross over”
