# Supplementary material for: A systematic review of decision aids that facilitate elements of shared decision-making in chronic illnesses: a review protocol
Source: Syst Rev. 2017 Aug 7;6:155. doi: 10.1186/s13643-017-0557-9 (PMC5545866; doi:10.1186/s13643-017-0557-9)
Supplement: Supplementary file 3 — Data to extract. (PDF 176 kb) [file 13643_2017_557_MOESM3_ESM.pdf]

Data to extract

**Publication**
Pilot RCT or RCT<br><br>1. Is the regarding publication a report of a RCT or a pilot RCT? (check one box)<br><br>0 pilot RCT<br><br>0 RCT
Name RCT<br><br>2. Acronym RCT:<br><br>3. Full name RCT:
Funding source(s) RCT<br><br>4. Name funder RCT:<br><br>5. Copy and paste the text under "Potential conflicts" here:
Authors<br><br>6. Authors (as follow: surname 1st author, first initial 1st author, second initial 1st author, … ; surname 2nd author, … ; … etcetera):
Title<br><br>7. Full title of publication:
Publication date<br><br>8. Publication year:
Journal<br><br>9. Full name of journal:
Language<br><br>10. The full-text article is written in (before translation by us or our colleagues) (check a box):<br><br>0 English<br><br>0 Spanish

0 German

0 Other, namely ………………………………………………………………………………………….

<u>Inclusion criteria</u>

11. Describe all inclusion criteria:

<u>Exclusion criteria</u>

12. Describe all exclusion criteria:

<u>Setting</u>

13. The trial setting is:

0 primary care

0 secondary care

0 tertiary care

0 both primary, secondary and tertiary care

0 both primary and secondary care

0 both primary and tertiary care

0 both secondary and tertiary care

0 other, namely …………………………………………………………………………

<u>Study population</u>

14. Total number of participants included:

15. Total number of participants analysed:

16. Condition (check one or more boxes):

0 suffering from a cardiovascular disease, namely (give type of CVD)

…….

0 suffering from diabetes mellitus, namely (give type of DM)

…….

0 suffering from a chronic respiratory disease, namely (give type of CRD)

………

0 other, namely ………………………………………………………………………………………….

17. Country where RCT took place:

18. Mean age and standard deviation:

19. Percentage males:

20. Percentage females:

Comparison(s)/control intervention(s)

21. Comparison(s)/control intervention(s) (check one or more boxes):

0 usual care (wait-list controls included)

0 active control, namely (give a description) ………………………………

Follow-up period

22. Duration of follow-up (in months):

23. Number of measurement moments (baseline and all follow-up):

Outcome(s) studied

24. Outcome(s) studied (check one or more boxes):

0 SDM outcomes

0 clinical outcomes

0 patient reported outcomes

DA characteristics

Name

25. Acronym DA:

26. Name DA:

Description DA

27. Decision to be made (describe as specific as possible):

28. Target group for which the DA meant to be used (describe as specific as possible):

0 cardiovascular disease patients, namely (give type of CVD)

……

0 diabetes mellitus patients, namely (give type of DM)

……

0 chronic respiratory disease patients, namely (give type of CRD)

……

0 other, namely

……

29. Setting in which the DA is meant to be used (check one or more boxes):

0 at the clinicians room

0 at the patient's home

0 at the waiting room

0 other, namely ……………………………………………………………………………………….

0 not stated

30. DA to be used by (check one or more boxes):

0 clinician, namely (check one or more boxes):

0 general practitioner

0 nurse

0 psychologist

0 clinical specialist, namely (explain kind of specialist) ……………………..

0 other, namely ………………………………………………………………………………

0 patient

0 relatives of the patient

0 other, namely ……………………………………………………………………………………….

31. DA delivery format (check one box):

0 online/digital

0 physical (on paper, etcetera)

0 partly online/digital and partly physical

0 both online/digital available and physical available

0 other, namely ………………………………………………………………………………………….

32. Training is needed for DA use:

0 yes

0 no

33. Describe how the DA looks like as specific as possible:

34. Describe how the DA is meant to be used as specific as possible:

<u>Funding source(s)</u>

35. Name funder DA:

<u>Availability</u>

36. DA is still available:

0 yes

0 no

37. Place of availability (check one or more boxes):

0 digital (if so, copy and paste the URL here ……………………………………………….)

0 physical (if so, describe where the DA is to be found here ………………………)

Effectiveness

<u>SDM outcomes (fill in per comparison of trial arms, per time span/interval and per outcome)</u>

38. Compared trial arms:

39. Time span/interval:

40. Type of SDM outcome:

0 decisional conflict → measurement instrument used: …………………………………………………………

0 knowledge → measurement instrument used: ……………………………………………………………………

0 patient participation in decision making → measurement instrument used: ……………………………

0 treatment decision (preference) → measurement instrument used: ……………………………………..

0 treatment satisfaction → measurement instrument used: ……………………………………………………..

0 decision satisfaction → measurement instrument used: …………………………………………………………..

0 conversation satisfaction → measurement instrument used: ……………………………………………….

0 risk expectations and perceptions → measurement instrument used: ……………………………………

0 consultation time → measurement instrument used: ……………………………………………………………..

41. In case of continuous outcomes: point-estimate/beta:

42. In case of continuous outcomes: p-value point-estimate/beta:

43. In case of continuous outcomes: 95%-CI point-estimate/beta:

44. In case of dichotomous outcomes: OR (if RR provided, then calculate OR)[1]:

45. In case of dichotomous outcomes: p-value OR:

46. In case of dichotomous outcomes: 95%-CI OR (if for RR provided, then calculate for OR)[2]:

47. In case of dichotomous outcomes: RR (if OR provided, then calculate RR)[3]:

48. In case of dichotomous outcomes: p-value RR:

49. In case of dichotomous outcomes: 95%-CI RR (if for OR provided, then calculate for RR)[4]:

<u>Clinical outcomes (fill in per comparison of trial arms, per time span/interval and per outcome)</u>

50. Compared trial arms:

51. Time span/interval:

52. Type of clinical outcome:

0 LDL cholesterol → measurement instrument used: …………………………………………………………..

0 HDL cholesterol → measurement instrument used: ………………………………………………………….

0 total cholesterol → measurement instrument used: ………………………………………………………

0 triglycerides → measurement instrument used: ……………………………………………………………….

0 blood pressure → measurement instrument used: …………………………………………………………..

0 smoking status → measurement instrument used: …………………………………………………………..

0 (maximal) oxygen uptake → measurement instrument used: ……………………………………….<br><br>0 glycaemic control → measurement instrument used: ……………………………………………………<br><br>0 Body Mass Index (BMI) → measurement instrument used: …………………………………………..<br><br>0 adherence → measurement instrument used: …………………………………………………………………<br><br>0 achieving treatment goals → measurement instrument used: ……………………………………..<br><br>53. In case of continuous outcomes: point-estimate/beta:<br><br>54. In case of continuous outcomes: p-value point-estimate/beta:<br><br>55. In case of continuous outcomes: 95%-CI point-estimate/beta:<br><br>56. In case of dichotomous outcomes: OR (if RR provided, then calculate OR)[1]:<br><br>57. In case of dichotomous outcomes: p-value OR:<br><br>58. In case of dichotomous outcomes: 95%-CI OR (if for RR provided, then calculate for OR)[2]:<br><br>59. In case of dichotomous outcomes: RR (if OR provided, then calculate RR)[3]:<br><br>60. In case of dichotomous outcomes: p-value RR:<br><br>61. In case of dichotomous outcomes: 95%-CI RR (if for OR provided, then calculate for RR)[4]:
<u>Patient reported outcomes (fill in per comparison of trial arms, per time span/interval and per outcome)</u><br><br>62. Compared trial arms:<br><br>63. Time span/interval:<br><br>64. Type of patient reported outcome:<br><br>0 quality of life → measurement instrument used: …………………………………………………………………………<br><br>0 health status → measurement instrument used: ………………………………………………………………………….<br><br>0 anxiety → measurement instrument used: …………………………………………………………………………………..<br><br>0 illness-related distress → measurement instrument used: ………………………………………………………<br><br>0 self-efficacy → measurement instrument used: ……………………………………………………………………………….<br><br>65. In case of continuous outcomes: point-estimate/beta:<br><br>66. In case of continuous outcomes: p-value point-estimate/beta:<br><br>67. In case of continuous outcomes: 95%-CI point-estimate/beta:

68. In case of dichotomous outcomes: OR (if RR provided, then calculate OR)[1]:

69. In case of dichotomous outcomes: p-value OR:

70. In case of dichotomous outcomes: 95%-CI OR (if for RR provided, then calculate for OR)[2]:

71. In case of dichotomous outcomes: RR (if OR provided, then calculate RR)[3]:

72. In case of dichotomous outcomes: p-value RR:

73. In case of dichotomous outcomes: 95%-CI RR (if for OR provided, then calculate for RR)[4]:

<u>SDM elements</u>

74. DA handles the situation diagnosis element:

0 yes → if so, describe as specific as possible how: …………………………………..

0 partly → if so, describe as specific as possible how: ………………………………..

0 no

75. DA handles the choice awareness element:

0 yes → if so, describe as specific as possible how: …………………………………..

0 partly → if so, describe as specific as possible how: ……………………………….

0 no

76. DA handles the option clarification element:

0 yes → if so, describe as specific as possible how: …………………………………..

0 partly → if so, describe as specific as possible how: ………………………………..

0 no

77. DA handles the harms and benefits discussion element:

0 yes → if so, describe as specific as possible how: …………………………………..

0 partly (also to be checked when harms and benefits of some, but not all, options are discussed) → if so, describe as specific as possible how: …………

0 no

78. DA handles the patient preferences deliberation element:

0 yes → if so, describe as specific as possible how: …………………………………..

0 partly → if so, describe as specific as possible how: ……………………………….

0 no

79. DA handles the decision making element:

0 yes → if so, describe as specific as possible how: …………………………………….

0 partly → if so, describe as specific as possible how: ……………………………….

0 no

[1]If OR is not presented, then calculate OR

[2]If 95%-CI for OR is not presented, then calculate 95%-CI for OR

[3]If RR is not presented, then calculate RR

[4]If 95%for RR is not presented, then calculate 95%-CI for RR
